# Supplementary material for: SCYN: single cell CNV profiling method using dynamic programming
Source: BMC Genomics. 2021 Nov 16;22(Suppl 5):651. doi: 10.1186/s12864-021-07941-3 (PMC8596905; doi:10.1186/s12864-021-07941-3)
Supplement: Supplementary file 1 — Additional file 1 The PDF file includes all the supporting materials for the manuscript [file 12864_2021_7941_MOESM1_ESM.pdf]

SCYN: Single cell CNV profiling method using dynamic programming
Supplementary File

Xikang Feng, Lingxi Chen,
Yuhao Qing, Ruikang Li, Chaohui Li,
and Shuai Cheng Li*

August 17, 2021

Contents

1 Supplementary Figures **2**

1 Supplementary Figures

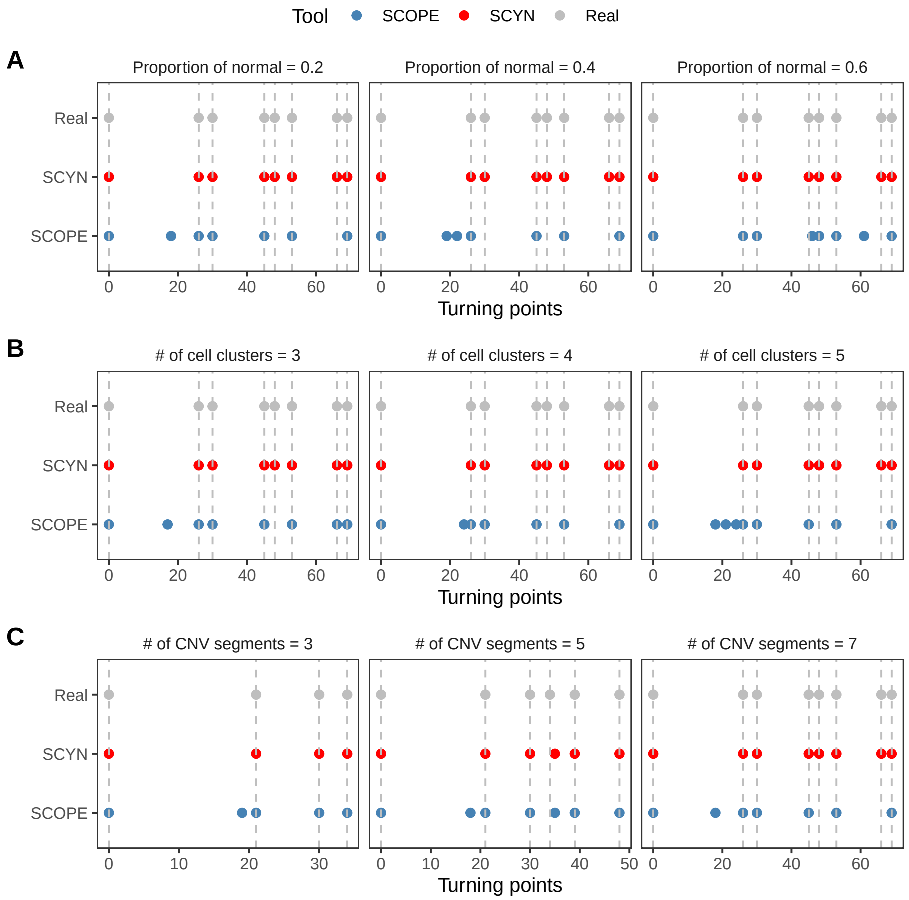

Figure S1: (A-C) The *in silico* experiments for CNV turning points detection with (A) different proportion of normal cells, (B) different number of cell clusters, and (C) different number of CNV segments, respectively. The x-axis lists the synthetic genome bins in order. The gray dot and vertical dashed line signifies the ground-truth turning points, while the red and blue dot refers to the turning point detected by SCYN and SCOPE, respectively.

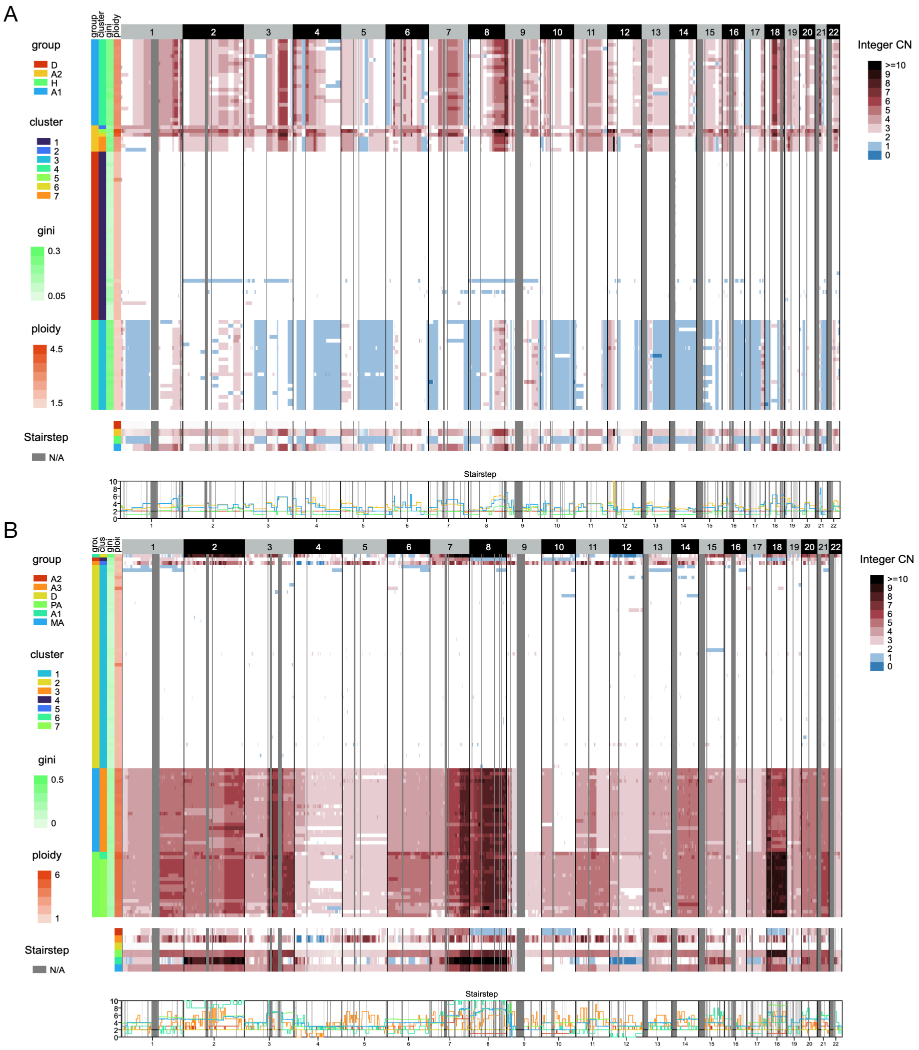

Figure S2: (A-B) Whole genome CNV profiles called by SCOPE on T10 and T16, respectively. From top to bottom, there are CNV heatmap, aggregated group CNV heatmap, and aggregated group CNV stairstep. The colorbar (blue, white, dark red) represents the value of copy number from 0 to ≥10, respectively. N/A denotes the missing of copy number in corresponding genomic region. The plots were visualized by scSVAS (https://sc.deepomics.org/demo-project/analyses/view).

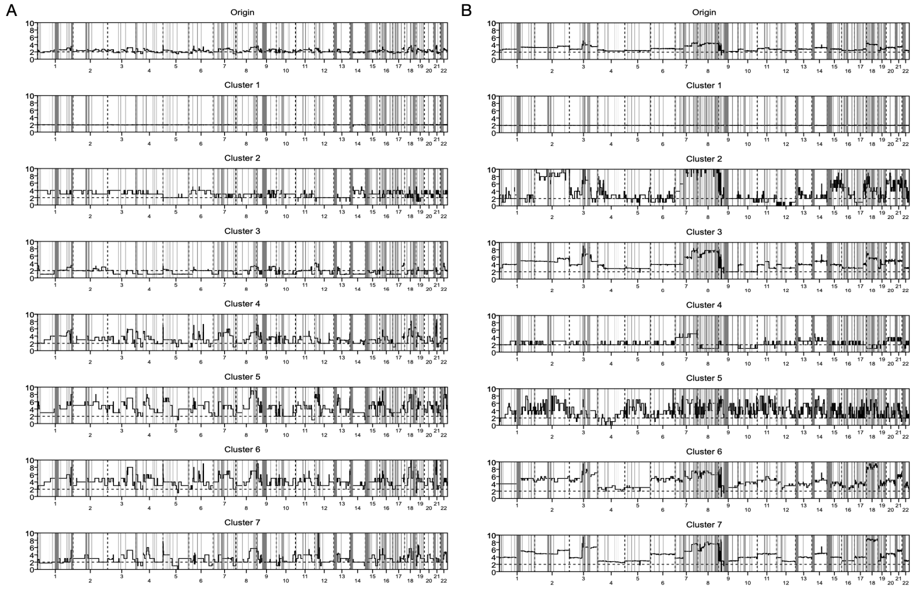

Figure S3: (A-B) The total (original) and subcluster-level collapsed ploidy plot of SCYN called CNV profiles on T10 and T16, respectively. Gray tile denotes the missing of copy number in corresponding genomic region. The plots were visualized by scSVAS (https://sc.deepomics.org/demo-project/analyses/ploidy_stairstep).

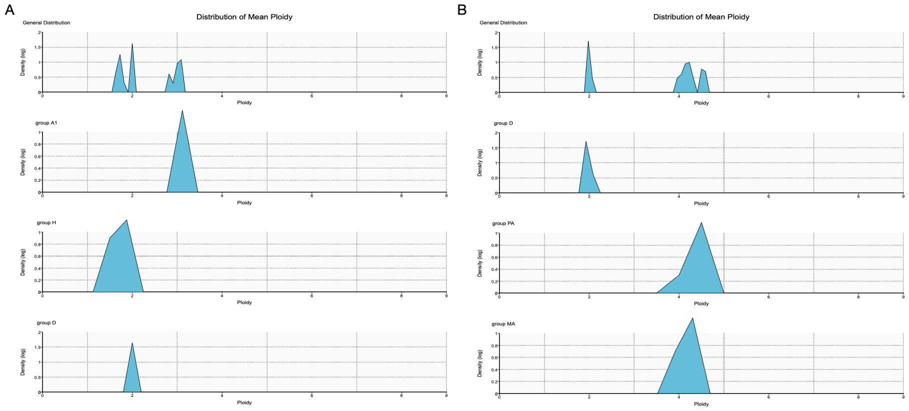

Figure S4: (A-B) The total and subgroup-level mean ploidy distribution of SCYN called CNV profiles on T10 and T16, respectively. The plots were visualized by scSVAS (https://sc.deepomics.org/demo-project/analyses/ploidy_distribution).

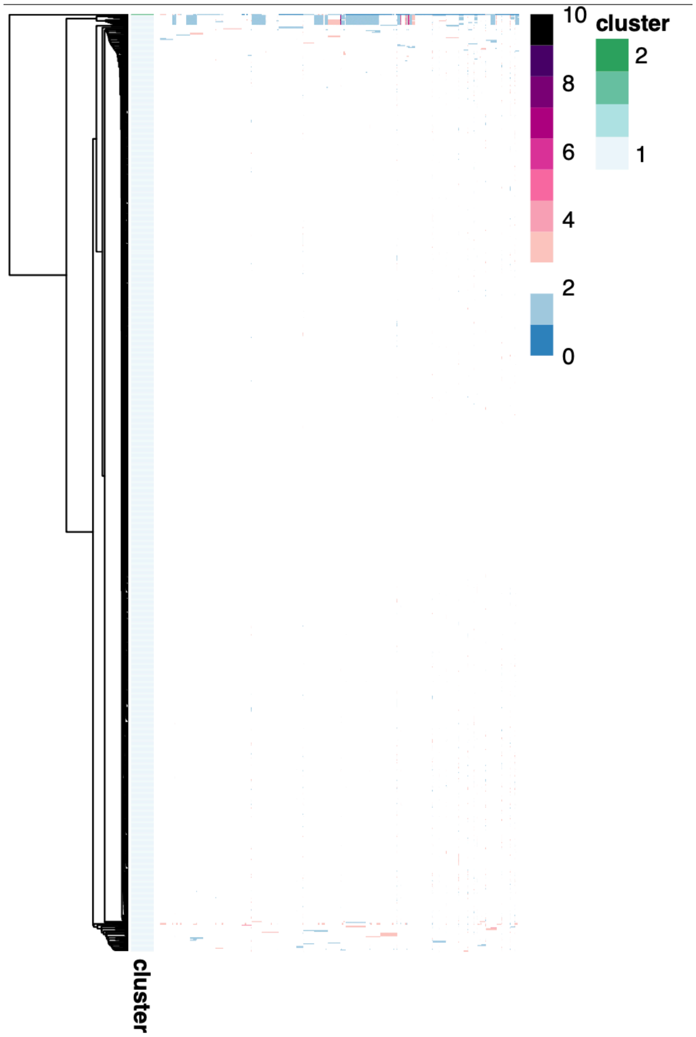

Figure S5: Whole genome CNV profiles called by SCYN on 10x 1% spike-in. The colorbar (blue, white, dark red) represents the value of copy number from 0 to ≥10, respectively.

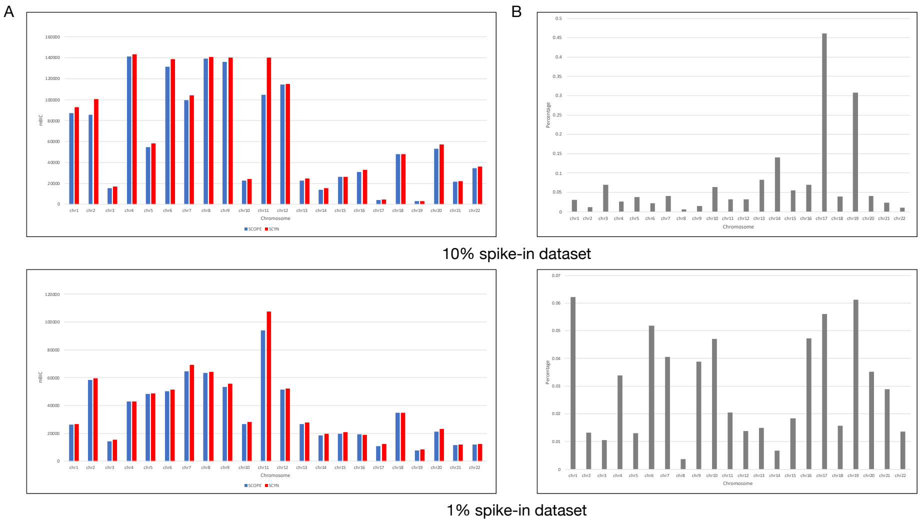

Figure S6: (A) SCOPE-mBIC of 10% and 1% spike-ins across all chromosomes generated by SCYN and SCOPE, respectively (Blue for SCOPE, red for SCYN). (B) The proportion of residual terms over SCOPE-mBIC across all chromosomes on 10% and 1% spike-ins, respectively.
